# Population genetic structure of *Wikstroemia monnula* highlights the necessity and feasibility of hierarchical analysis for a highly differentiated species

**DOI:** 10.3389/fpls.2022.962364

**Published:** 2022-10-07

**Authors:** Chaoqiang Zhang, Yiwei Tang, Defeng Tian, Yanyan Huang, Guanghui Yang, Peng Nan, Yuguo Wang, Lingfeng Li, Zhiping Song, Ji Yang, Yang Zhong, Wenju Zhang

**Affiliations:** ^1^ College of Life Sciences and Engineering, Hexi University, Zhangye, China; ^2^ Ministry of Education Key Laboratory for Biodiversity Science and Ecological Engineering, Institute of Biodiversity Science, Fudan University, Shanghai, China; ^3^ Institute for Preservation and Conservation of Chinese Ancient Books, Fudan University, Shanghai, China

**Keywords:** delta K, optimal K, hierarchical STRUCTURE analysis, population structure, *Wikstroemia monnula*

## Abstract

Population genetic structure can provide valuable insights for conserving genetic resources and understanding population evolution, but it is often underestimated when using the most popular method and software, STRUCTURE and delta K, to assess. Although the hierarchical STRUCTURE analysis has been proposed early to overcome the above potential problems, this method was just utilized in a few studies and its reliability needs to be further tested. In this study, the genetic structure of populations of *Wikstroemia monnula* was evaluated by sequencing 12 nuclear microsatellite loci of 905 individuals from 38 populations. The STRUCTURE analysis suggested the most likely number of clusters was two, but using multi-hierarchical structure analysis, almost every population was determined with an endemic genetic component. The latter result is consistent with the extremely low gene flow among populations and a large number of unique cpDNA haplotypes in this species, indicating one level of structure analysis would extremely underestimate its genetic component. The simulation analysis shows the number of populations and the genetic dispersion among populations are two key factors to affect the estimation of K value using the above tools. When the number of populations is more than a certain amount, K always is equal to 2, and when a simulation only includes few populations, the underestimation of K value also may occur only if these populations consist of two main types of significantly differentiated genetic components. Our results strongly support that the hierarchical STRUCTURE analysis is necessary and practicable for the species with lots of subdivisions.

## Introduction

Revealing the correct population genetic structure (the “true” number of clusters) of a species can provide the scientific basis for formulating conservation strategies and sustainable utilization of genetic resources of the species (Worth et al., 2014; Wang et al., 2016; Wu et al., 2020). STRUCTURE (Pritchard et al., 2000) has been the most widely used software to detect hidden genetic structures; it implements a Bayesian clustering algorithm to simultaneously assign all individual samples to genetic clusters without the need for predefined spatial or genetic population information by calculating maximum likelihood in searching for the most optimal K value. But in many studies using STRUCTURE, there was no clear maximum likelihood, the key to identify the optimal K value. Thus, the Delta K method, a statistic based on the second-order rate of change of Ln Pr (X|K), was introduced (Evanno et al., 2005) and has been widely used to infer the most likely number of genetic clusters. However, Janes et al. (2017) reviewed 1264 studies that used STRUCTURE to explore population subdivision, and found that in more than half (54%, 443/822) of studies used delta K, K was equal to 2 (K=2) even when there were more subdivisions in the samples. The authors concluded that many studies that identified K=2 likely underestimated population genetic structure when using Ln Pr (X|K) and/or delta K.

Hierarchical STRUCTURE analysis may be one possible approach to solve the problem of underestimation of the optimal K value when using delta K method. For STRUCTURE version 2.1 (Pritchard and Wen, 2003), the authors recommended the additional practice of performing subsequent STRUCTURE runs on previously identified clusters, to detect further patterns of genetic structure (Pritchard and Wen, 2003). Also, Evanno et al. (2005) concluded that Delta K was likely to detect the uppermost level of genetic structure patterns, and subsequent hierarchical analysis should be carried out. In fact, before the delta K method was proposed, Rosenberg et al. (2002) introduced the concept of reanalyzing subsets of data, although their reanalysis was performed based on geographic subdivisions rather than clusters. However, as Janes et al. (2017) reported, only 11% of studies performed the hierarchical analysis to fully explore population subdivisions. It is important to test the reliability of this method, and whether the population genetic structure is underestimated or overestimated.

Here, we chose *Wikstroemia monnula* as the research material to examine the above problems. *W. monnula* Hance (Thymelaeaceae) is a deciduous shrub endemic to southeast China. Populations of this species are scattered on isolated rock walls, mountain tops, cliffs, or slopes, at elevations ranging from c.200-1365 m above sea level (**Table S1**). As a rock wall shrub, it is generally found in narrow strips. It has long been used as an important raw material for precious Chinese traditional handmade paper. However, due to specific habitats, low population density, and excessive cutting by humans, its natural populations have declined during the past decades and are threatened with extinction. In our previous study, using chloroplast DNA fragments, we found that *W. monnula* had high genetic diversity and specific cpDNA haplotypes in almost every population of it (Zhang et al., 2022).

In the present study, we collected 38 populations throughout the distribution range of *Wikstroemia monnula* and used nuclear EST-SSR markers. The aim was to analyze the subpopulations of *W. monnula* for getting the “true” number of clusters of genetic structure and to explore the reliability of the hierarchical analysis, characterizing the extent and possible causes of the underestimation of the number of genetic clusters.

## Materials and methods

### Population sampling

We collected leaf materials of 905 individuals from 38 populations of *Wikstroemia monnula* across its geographic range. Each population of *W. monnula* contained 9 to 33 individuals. All sampled populations are located in the mountainous areas of southeast China (extending between ≈ 22° N and 30° N, including Nanling, Wuyi, and parts of Luoxiao and Xuefeng Mts.) (**Figure 6**; **Table S1**). Fresh leaves of plants growing at least 15 m apart were collected and stored in silica gel until DNA extraction. Voucher specimens were collected for each population and deposited in the Herbarium of the School of Life Science, Fudan University, Shanghai, China (FUS). The information on latitude, longitude, and altitude of each *W. monnula* population was recorded using an eTrex Global Positioning System (Garmin) (**Table S1**).

### Total RNA extraction and developments and validation of EST-SSR markers

Total RNA was extracted from the leaves and was sequenced by the HiSeqTM 2500 NGS platform. ‘Clean reads’ were assembled using Trinity software (v2.0.6) (Grabherr et al., 2011). The transcripts were clustered and obtained final unigenes (Pertea et al., 2003).

The software MISA was used to search the SSR sites (Simple sequence repeats) in all unigenes (Haas et al., 2013). The configuration parameters are as follows: the minimum repetition times of di-, tri-, tetra-, penta-, and hexa-nucleotide (motifs) repeats were 7, 6, 5, 4, and 4, respectively, and the maximum difference between two SSRs setting at 100 bp. EST-SSR primers were designed based on the flanking conserved sequences of SSR locus using Primer 3.0 software (Untergasser et al., 2012), Following characteristics: (1) primer length ranging from 18-26 bp with an optimum 25 bp; (2) an annealing temperature ranging from 56°C to 62°C, with a difference of less than 2°C between forward and reverse primers; (3) an amplification product sized between 126 and 428 bp; and (4) a GC content ranging from 40 to 60 with an optimum of 50%. In total, 100 EST-SSR primers were randomly selected for synthesis ((synthesized by Sangon Biotech (Shanghai) Co., Ltd.)) and were screened by eight *Wikstroemia monnula* samples (different populations). Finally, 12 pairs of EST-SSR primers (**Table S2**) with clear bands and obvious polymorphism were selected for genetic structure analysis of 38 populations of *W. monnula*.

### DNA extraction, amplification, and microsatellite genotyping

Total genomic DNA was extracted using a plant genomic kit (Tiangen Biotech, Beijing, China) following the manufacturer’s manual. Twelve polymorphic nuclear microsatellites were screened by PCR amplification followed by denaturing polyacrylamide gel electrophoresis (**Table S2**). Selected markers were labeled at the 5’ end of the forward primer using one of the three fluorescent dyes: ROX, JOE and 6-FAM (5’ adapter sequence M13:5’CACGACGTTGTAAAACGAC-3’) (Sangon Biotech (Shanghai) Co., Ltd.). PCR was performed in a volume of 10 μL containing 1.5 μL of template DNA (ca. 50 ng), 2.9 μL of 2×Taq PCR MasterMix (Tiangen, Shanghai, China), 0.04 μL of forward primer (10 mM) + 0.36 μL M13 (10 mM), and 0.4 μL reverse primers (10 mM) and 4.94 μL of ddH_2_O. The PCR procedure was as follows: initial denaturation at 94°C for 3 min, followed by 35 cycles of 30 s at 94°C, 40 s at 56-62°C, and 45 s at 72°C, with an additional extension at 72°C for 10 min. The PCR products were detected using an ABI3730XL DNA Analyzer (Shanghai Mapbioo Biotechnology Co., LTD, Shanghai, China), and alleles were scored manually using GeneMapper (version 3.7.0).

### Data analysis

For the nSSR dataset, we first conducted the neutral test and the test for deviations from Hardy-Weinberg equilibrium (HWE). We used STRUCTURE v.2.3.4 (Pritchard and Wen 2003) to infer genetic structure and define the number of clusters in the dataset. We performed 12 runs at each value of the fixed parameter K (the number of clusters) ranging from one to 12. Each run consisted of 500,000 replicates of the MCMC after a burn-in of 100,000. All other parameters were set to default values. The most likely number of homogeneous clusters was assessed by standardizing the second-order rate of change of the mean likelihood of K (i.e. Delta K, Evanno et al., 2005). The online application STRUCTURE HARVESTER (Earl and Vonholdt, 2012) was used to visualize the structure results.

For the hierarchical structure analysis, the top level of hierarchical structure analysis result was analyzed for the second level of hierarchical structure analysis, and so on until all the cluster results could no longer be divided. As with the top level of hierarchical structure analysis, the most likely number of homogeneous clusters was assessed by standardizing the second-order rate of change of the mean likelihood of K (i.e. Delta K, Evanno et al., 2005), using the online application STRUCTURE HARVESTER (Earl and Vonholdt, 2012).

To estimate the effect of population number on optimal K value, we randomly selected populations numbered 4, 6, 8, 10, 12, 16, 20, and 30 from 38 populations of *Wikstroemia monnula* and ran 10 iterations at every level. Each population was sampled only once in each sample recombination. According to the above method, the genetic structures of 80 randomly sampled population combinations were analyzed, the optimal K value of each combination was determined, and the distribution of K value was calculated. In order to estimate the effect of genetic differentiation among populations on K value, additional 50 stimulations with 4 populations were conducted following the above method.

The gene flow (*N*m) and Nei’s genetic distance (Nei et al., 1983) were estimated in GENALEX 6.5 (Peakall and Smouse, 2006). The pairwise genetic distances (*F*_ST_) among populations were calculated using Arlequin V.3.11 (Excoffier et al., 2005). The unweighted pair-group method with arithmetic means (UPGMA) tree based on the Nei’ genetic distance matrix was constructed using NTSYS-pc, version 2.11 a (Rohlf, 2000). A principal coordinate analysis (PCoA) was constructed using GENALEX v 6.5 (Peakall and Smouse, 2012) based on the pairwise *F*_ST_ matrix and visualized with the R package “ggplot2” (Villanueva and Chen, 2019).

Data of chloroplast haplotypes of *Wikstroemia monnula* were obtained from our previous work (Zhang et al., 2022).

## Results

### Gene flow and genetic differentiation based on SSR data

**Figure 1:**
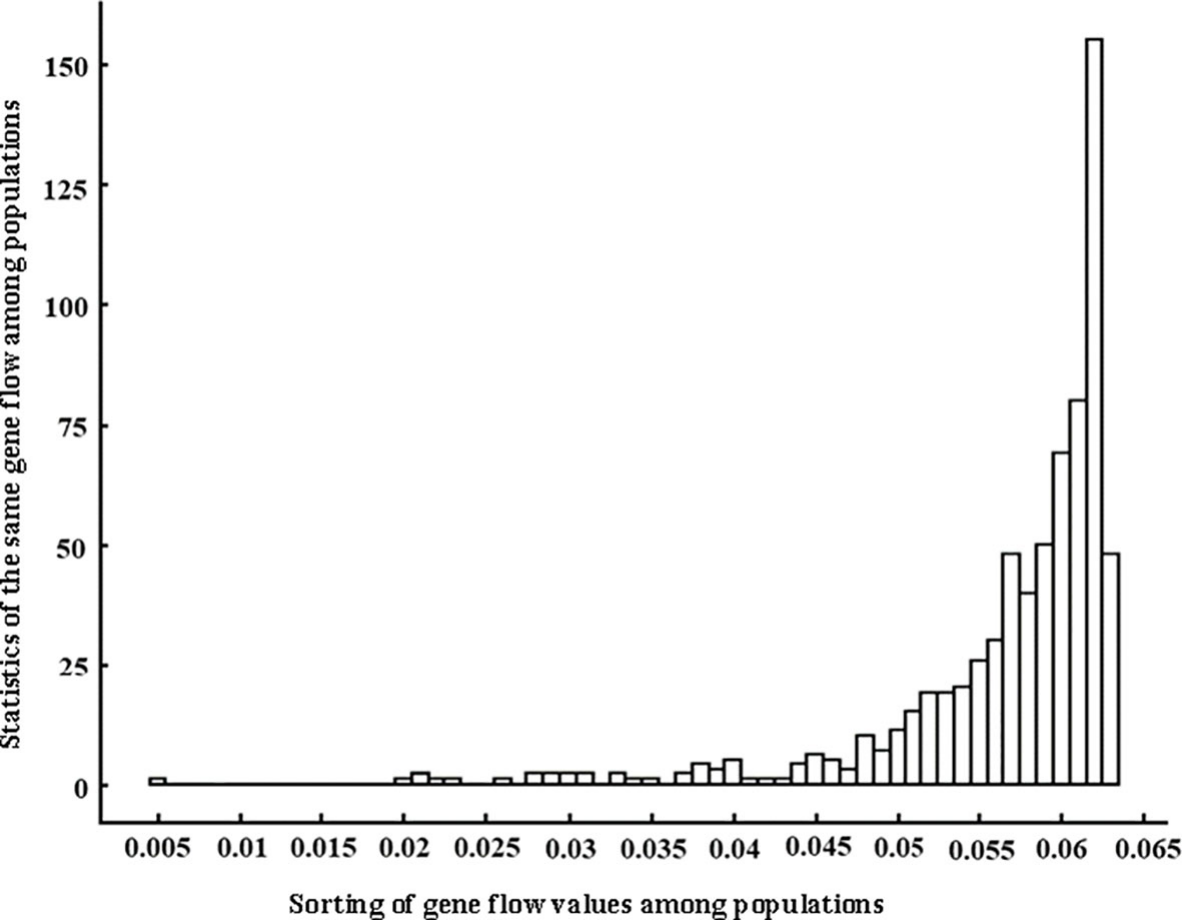
Distribution of gene flow values among 38 populations of *Wikstroemia monnula* based on twelve microsatellite loci. The horizontal axis is sorted in gene flow value orders from low to high, and the vertical axis is total number of gene flow with the same value.

The gene flow among 38 populations of *Wikstroemia monnula* was from 0.005 to 0.063 (**Figure 1**; **Table S3**). This is shown in **Figure 1**, with the gene flow among most populations being about 0.06, and 0.063 being the highest, accounting for 22%. The differentiation index (*F*st) among 38 populations was between 0.372 and 0.743, with an average of 0.474. According to Balloux and Lugon-moulin (2002), the *F*st value greater than 0.25 indicated a high level of population differentiation; hence, a high differentiation occurred among the populations of *W. monnula* characterized in the present study. In the AMOVA, 12.66% (*P* < 0.001) of the total EST-SSR variation was distributed among groups, 31.72% was explained by variation among populations within groups, and 55.61% was apportioned within populations. These results showed genetic variation was high within populations, among groups, and among populations.

### Genetic clustering based on SSR data by hierarchical analysis

**Figure 2:**
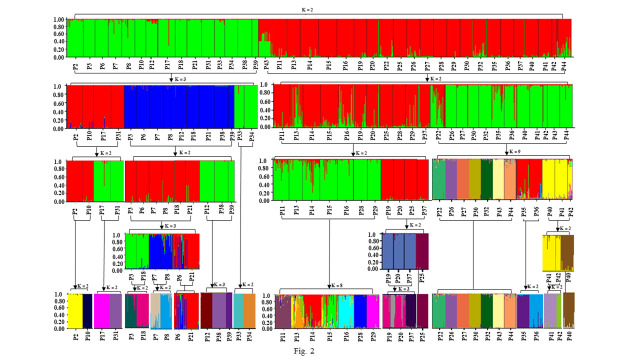
Progress of five hierarchical levels analysis of 905 individuals from 38 populations of *Wikstroemia monnula* using STRUCTURE based on twelve microsatellite loci. The optimal K based on the delta K (ΔK) methods for every hierarchical analysis was shown. All structure analysis were marked by black arrows, the populations for each analysis were labeled using bracket, and the K value indicated the optimal K for each analysis. The color of the STRUCTURE plot in the fourth and fifth hierarchy has been adjusted to avoid confusion of different genetic components.

The delta K analysis including 905 individuals from 38 populations suggested the most likely number of clusters was two (K=2, **Figure 2**). When these two clusters were analyzed separately, cluster 1 (including 15 populations) was divided into three sub-groups (**Figure 2**), each of which was further subdivided into the third level of hierarchical structure. In the third level analysis, P33 and P34 population samples were first clustered into two separate groups (**Figure 2**), and the remaining populations required the fourth (P2, P10, P17, and P31) and fifth (P3, P6, P7, P18, and P21) hierarchical analysis. Total individuals of 15 populations in cluster 1 were finally divided into 15 distinct genetic components, and each population almost consists of a single genetic component, but P6 and P21, P7 and 8. (**Figure 2**).

Cluster 2 (including 23 populations) produced two sub-groups (**Figure 2**) when analyzed in isolation (K=2). When the third STRUCTURE analysis was conducted on the two sub-groups, the group1 (populations P11, P13, P14, P15, P16, P19, P20, P25, P28, P29, and P37) was further divided into two sub-groups (K=2), The former sub-group (P11, P13, P14, P15, P16, P28, and P29) was further divided into eight sub-groups (K=8), indicating very strong population differentiation. And the group2 (P22, P26, P27, P30, P32, P35, P36, P40, P41, P42, P43, and P44) was divided into nine sub-groups (K=9). The remaining populations continued to be carried out to the fourth (P40, P41, and P42) and fifth (P19, P20, P25, and P37) hierarchical STRUCTURE analysis (**Figure 2**). After the fifth STRUCTURE analysis, no further clustering was detected. The final results showed that 68.42% of populations (26/38) had their own unique genetic component (**Figure 2**).

### UPGMA and PCoA clustering based on genetic distance

**Figure 3:**
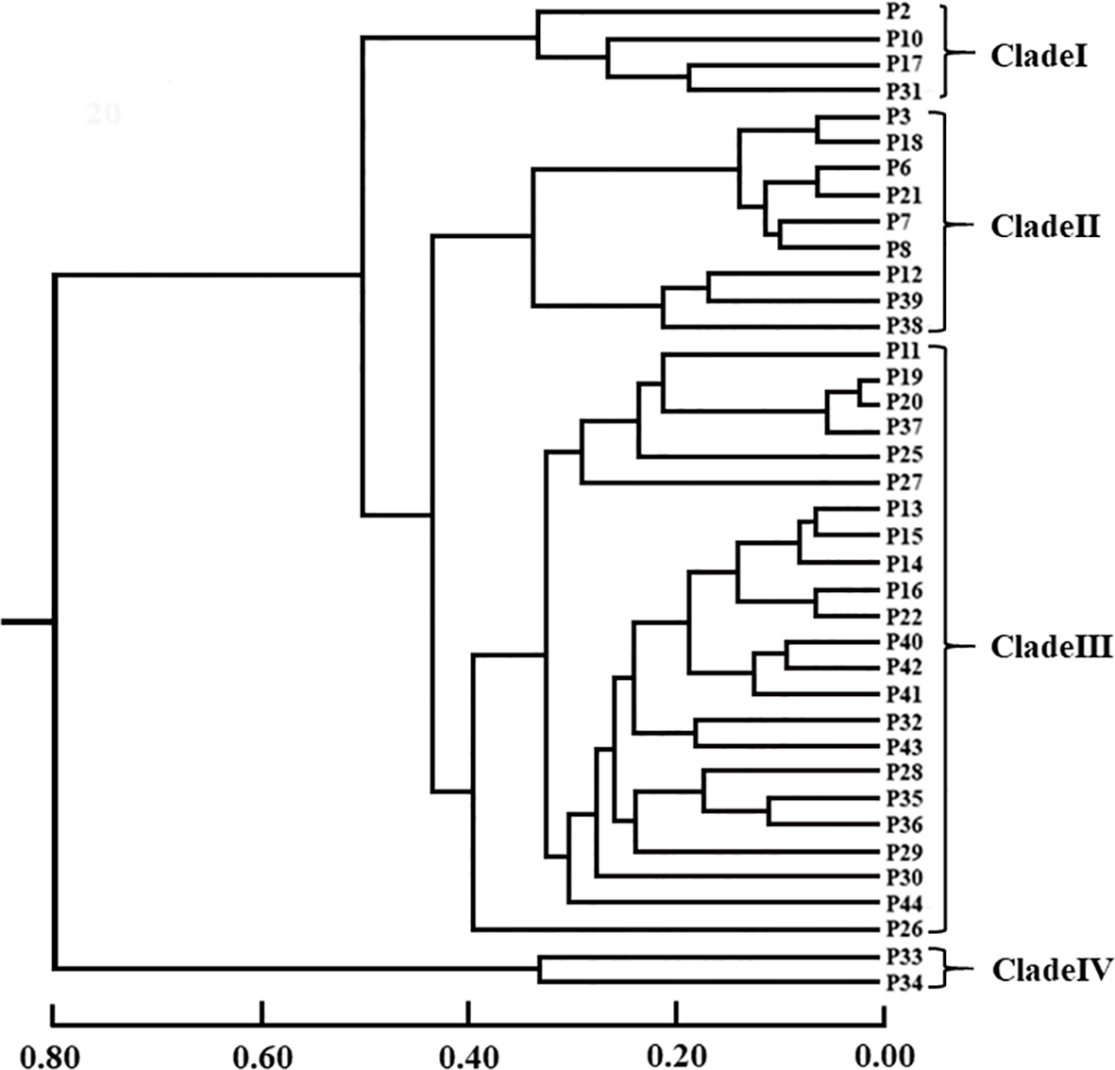
The UPGMA dendrogram of 38 populations of *Wikstroemia monnula* based on 12 SSR markers developed in this study. Scale bar represents the genetic distance. The brackets on the left marked four main clades, in which clade II and clade Ш include two subclades, respectively.

UPGMA using Nei’s genetic distance method was shown in **Figure 3**. Results found that 38 populations were clustered obviously into 4 groups. P33 (Wenzhou population) and P34 (Yandang Mountain population) are grouped together, being obviously different from other populations (Clade IV, **Figure 3**). Clade I included P2, P10, P17, and P31. Clade II included 9 members, which could be divided into two sub-groups, viz., the one containing P3, P6, P7, P8, P18, and P21, and the other containing P12, P38 and P39. Clade III was the largest group, consisting of 23 populations, and could be further divided into three sub-groups. Among them, P26 consisted of a sub-group. It is noteworthy that even the four populations living far apart spatially (P2, P10, P17, and P31) clustered into one group (clade I). The two of these populations (P2 and P10) are distributed in the eastern part of Sanqing Mountain population (P2) in Jiangxi province and Jiangshan City population (P10) in Zhejiang Province, whereas the other two populations (P17 and P31) are located in the western part of Huaping National Nature Reserve: population P17 in Guangxi province and Yuanbao Mountain and population P31 in Rongshui county, Guangxi province.

**Figure 4:**
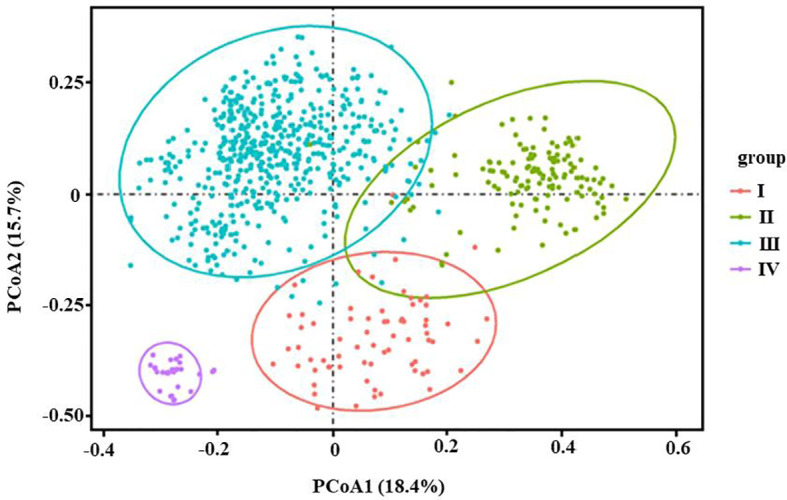
Principal coordinate analysis (PCoA) of 38 populations of 905 individuals of *Wikstroemia monnula* based on Nei’s unbiased genetic distances. Horizontal (PCoA1) and vertical (PCoA2) scales represent the first and second principal axes of variation respectively (PCoA1 and PCoA2 that explain the most variations among populations). Here PCoA1 represents a large 18.4% of variation, the PCoA2 represents 15.7% of variation, and 38 populations of *W. monnula* could be divided into four different groups. Group I: P2, P10, P17 and P31 (105 individuals); Group II: P3, P6, P7, P8, P12, P18, P21, P38 and P39; Group IV P33 and P34; group III: other 23 populations.

In this study, PCoA analysis was also conducted on 905 individuals based on genetic distance matrix, and the results were shown in **Figure 4**. The horizontal axis explained 18.4%, whereas the vertical axis explained 15.7%. It can be seen from **Figure 4** that 905 individuals clustered into four distinct groups, Group I (red) corresponding to four populations (P2, P10, P17, and P31) of 105 individuals, Group II (green) had nine populations (P3, P6, P7, P8, P12, P18, P21, P38, and P39) of 197 individuals, Group IV (purple) had two populations from Wenzhou (P33) and Yandang Mountain (P34) of 42 individuals, and the remaining 531 individuals from 23 populations constituted Group III (light blue). The results of the UPGMA dendrogram and Principal coordinate analysis were basically the same.

### Change in optimal K values with an increase in population number

**Figure 5:**
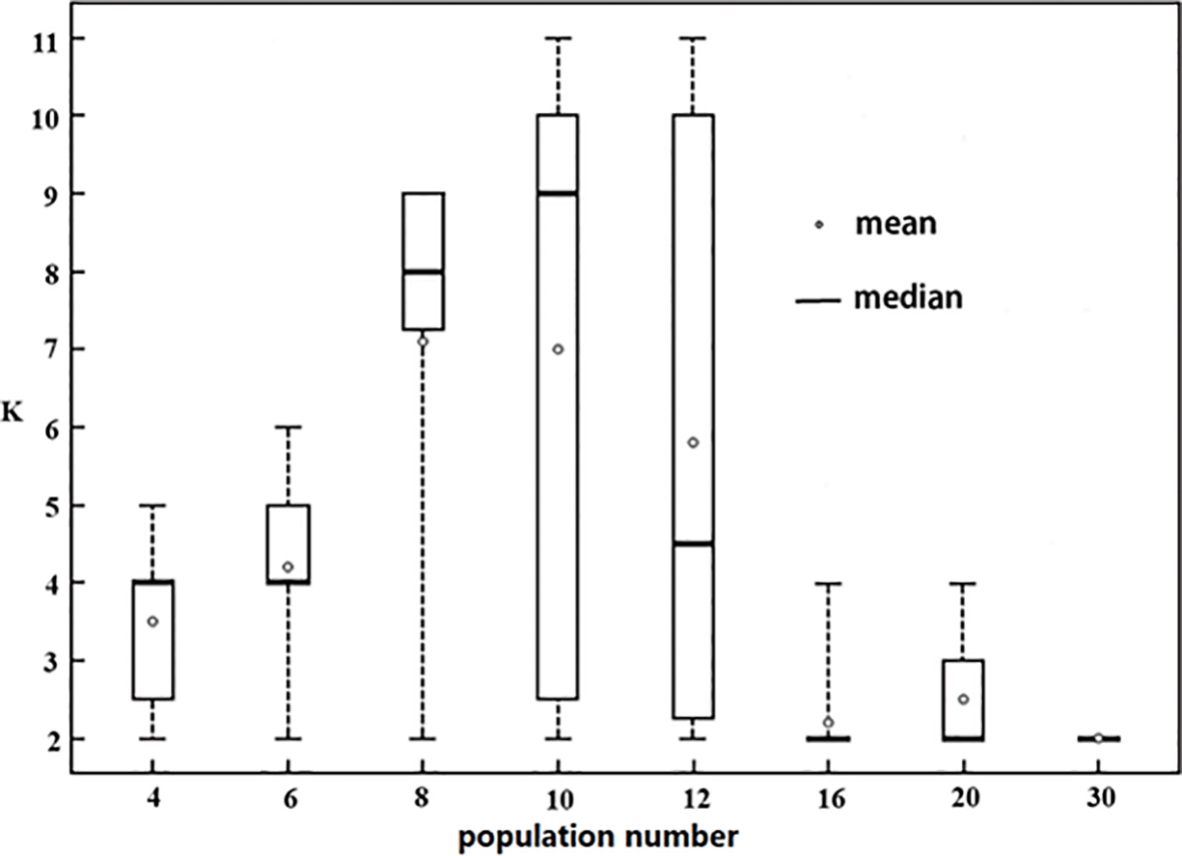
Change of optimal K values with the increase of sampling population number. The number on the horizontal axis is the number of populations randomly selected from 38 populations, the vertical axis indicates optimal K value obtained in STRUCTURE analysis. At every level of population number, 10 random samples were conducted without repetitive populations in every sample, and the range, the maximum, the minimum, the mean value, and the median of optimal K were shown.

Genetic structures of eighty randomly sampled groups from 38 populations were analyzed using the delta K method in this study (**Figure 5**). Among them, 35 groups had K=2. With the increase in population numbers, both the mean value and median value of K showed a trend of rising first and then falling; the maximum K value occurred when the population numbers equaled 10. When the population numbers exceeded 16, the median K value was 2, and the mean value was also very close to 2; when the population numbers were less than or equal to 10, all the median values were close to the population numbers. Moreover, we found that regardless of population number, there would always be some result K equaling 2.

### Change in optimal K values with genetic differentiation among populations

The results of 60 simulations with 4 populations but including different clades and/or subclades were shown in **Table 1**. Of 60 simulations, 23.33% (14) of tests obtained K=2. When the simulation sample consisted of populations from 3 or 4 clades, there was no K=2 (15 simulation) (0/15); but when those consisted of populations from 2 clades, 58.82% (10/17) of them obtained K=2. If we divided Clade II and Clade III in UPGMA into two obvious subclades, respectively, we noticed that when 4 populations were from 2 clades but included 3 subclades, the rate of K=2 decreased sharply (9.52%, 2/21).

**Table 1:** Statistics of K values and *F*st of 60 simulations with 4 random populations and different clades or subclades using STRUCTURE and the delta K (ΔK) methods.

Clade numberStatistics values	3 Clades or 4 clades	2 Clades but 3 subclades	2 Clades	1 Clade
Number of simulations	15	21	17	7
Number of simulations with K=2	0	2	10	2
Mean of K values	4.13	3.81	2.76	3.14
Mean of *F*st	0.58	0.51	0.46	0.38
Standard deviation of *F*st	0.15	0.13	0.15	0.09

The clades or subclades are shown in **Figure 3** (the UPGMA dendrogram).

## Discussion

### Reliability of hierarchical STRUCTURE analysis

**Figure 6:**
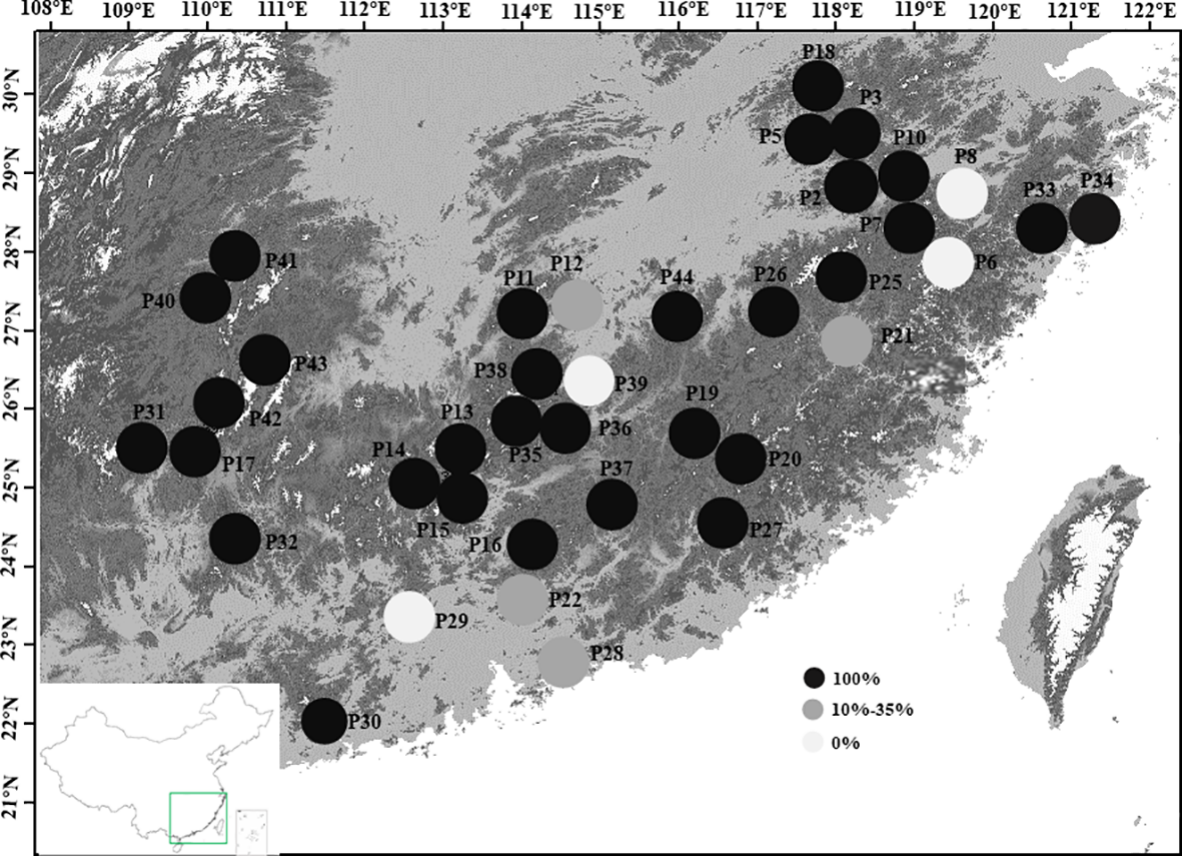
Proportion of private cpDNA haplotypes in 38 sampled populations of *Wikstroemia monnula.* The private haplotype is endemic to one local population and not to be shared by any other population. Haplotype information was from Zhang et al., 2022.

In this study, we found that *Wikstroemia monnula* has extremely low gene flow among 38 populations (**Table S3**, with an average of 0.051), and a strong genetic differentiation (*F*st, with an average of 0.474). However, when we used STRUCTURE 2.3.4 to analyze the structure of this species, the optimal K value was observed at K=2 with maximum delta K, with all individuals divided into two clusters (**Figure 2**). This result also contradicts the above extremely low gene flow and the highly specific cpDNA haplotypes in various populations (**Figure 6**, Zhang et al, 2022). Janes et al. (2017) showed that when K value was 2, population genetic structures were often underestimated by the STRUCTURE analysis; hence, they strongly recommended hierarchical STRUCTURE analysis. After five levels of hierarchical analysis, 39 genetic components were revealed from 38 populations (**Figure 2**), more than 50% of populations consisted of a single specific genetic component. If this value (39) is correct, then the true genetic component would be extremely underestimated when we selected the K value based only on the first level of STRUCTURE analysis.

We think that the hierarchical analysis results reported above are correct for the following reasons. (1) The gene flow among the 38 populations of the target species was extremely low (0.005 to 0.063), and when gene flow is far below 1, it was difficult for populations to share the same genetic components (Stöcklin et al., 2009). (2) Even in the third level of hierarchical STRUCTURE analysis, some populations were far apart in geographical space (e.g. P2 and P31) but still had the same genetic constitution, which was difficult to explain. However, after the fourth level of hierarchical STRUCTURE analysis, it was revealed that the two populations specified above did have completely different genetic components (**Figure 2**), indicating a necessity to conduct the full hierarchical STRUCTURE analysis. (3) The results obtained by the fifth level of hierarchical analysis were highly consistent with the information obtained by cpDNA haplotypes. In the previous study (Zhang et al., 2022), 50 haplotypes (94.34%) were unique to only one local population, and. 30 of 38 (78.9%) populations only consisted of unique haplotypes, meaning there was no seed flow among most populations of *Wikstroemia monnula* (**Figure 6**). (4) Finally, the pollination characteristics and the habitat of the species are not conducive to gene flow. Observations in the field revealed this species grows in fragmented habitats on rock walls, and different populations are often obstructed by large areas of vegetation, which can be an obstacle to pollen dispersal. In addition, both ants and *Bombus* spp. are likely to be pollinators of *W. monnula*, and their foraging behavior is limited to the local area. These reasons would promote differentiation among populations and make very close populations have different genetic components. The above analysis indicated that the results obtained by the hierarchical STRUCTURE analysis were representative of the real population structure.

Is it possible to overestimate population genetic structure? That is, the hierarchical analysis of the same genetic component produces new genetic types. In the study on *Retropinna spp*, Hughes et al. (2014) found that the optimal K of the four populations was still equal to 1 after the third-level hierarchical analysis. In the present study, after the third analysis, many populations (e.g. P19, P20, P25, and P37 in **Figure 2**) still shared the same genetic component, we conducted the fourth and the fifth analysis, and P25 and P37 showed specific genetic components; however, P19 and P20 populations still had the same genetic constitution. After we conducted six hierarchical structure analyses of these two populations, no additional genetic components were identified. This result and Hughes et al.’s study showed that if populations are not composed of several genetic components, more hierarchical STRUCTURE analyses are unable to find more genetic components (i.e. overestimate genetic components). These results supported each population of *W. monnula* containing its specific genetic component and showed that the full hierarchical STRUCTURE analysis is not only necessary but also reliable.

### Effects of population number on K value

Does the number of populations have an effect on the optimal K value? Some simulations suggested that the species with many subpopulations had a large impact on STRUCTURE analysis (Evanno et al., 2005; Earl and Vornholdt, 2012; Janes et al., 2017). In the present study, we estimated the effect of population sample combinations on the optimal K value, and found that population number had a significant effect on genetic structure (**Figure 5**). From four to 30 populations, K=2 occurred, but in different proportions. These findings suggest that when the population number is not large, the STRUCTURE analysis tends to yield reasonably good estimates of K value, but may still underestimate the K value. Especially, when the population number was more than 16, the mean value of K was less than 3 (far lower than the population number, and far from reflecting the population subdivision), indicating that the genetic structure of most sample combinations was seriously underestimated.

Generally, no matter how many randomly sampled populations were included in the STRUCTURE analysis, population genetic structure (K value) may be underestimated. However, the population number of analysis has a significant impact on the extent of underestimation of the K value; with an increase in the sampling population number, the optimal K value would be underestimated more strongly and more frequently. This result indicates that the hierarchical structure analysis is necessary regardless of whether the population number is large or small. But when analyzing more populations, the necessity of the hierarchical structure analysis is even stronger.

### The effect of population genetic differentiation on K value

Verity and Nichols (2016) suggested that genetic dispersion (discrete populations) may be one of the reasons for underestimation. In this study, 60 stimulations only including 4 populations supported the above hypothesis. These results demonstrated genetic dispersion indeed affects STRUCTURE analysis even if the population number is very small. Especially, when a population group consists of two kinds of significantly differentiated genetic components or clades, the STRUCTURE analyses may tend to calculate the top level of hierarchical structure. This trend was even more obvious with the increase in population number. Of 30 simulations containing 16 or more random population samples, 23 of them (76.67%) obtained K=2 though they also included members from 3 or more clades in the UPGMA dendrogram. We noticed that the above 23 simulations of K=2, 20 contain the member of Clade IV (P33 and P34), which is obviously different from the other clades (**Figure 3**; **Figure 4**), with the highest mean genetic distance from other populations (0.7077 and 0.7434, respectively). There being an obvious hierarchical genetic structure may be the most important reason for K=2 when using Structure software to infer the optimal K of populations, which was likely to hugely underestimate the true genetic components of species.

## Conclusions

The study demonstrated a very complex population genetic structure of *Wikstroemia monnula*, featuring low gene flow among populations and extremely high population differentiation, with almost every population having its own unique genetic component. The usual STRUCTURE analysis, which obtained the optimal K=2, extremely underestimated the population genetic structure of *W. monnula*. Larger population numbers and more complex hierarchical genetic differentiation were two important factors to result in the underestimation of K value and the optimal K=2. For revealing the true population genetic structure, hierarchical structure analysis is necessary and practicable for species with highly subdivided populations.

## Data availability statement

The datasets presented in this study can be found in online repositories. The names of the repository/repositories and accession number(s) can be found below: https://www.ncbi.nlm.nih.gov/, accession number PRJNA379203 (MV713810-MV713826, MV713828-MV713841, MV714410-MV714485).

## Author contributions

W-JZ, Y-GW, L-FL, Z-PS, JY, YZ and G-HY conceived the study. C-QZ, Y-YH and PN collected the materials. C-QZ, Y-WT and D-FT performed the statistical analyses and C-QZ wrote the first draft of manuscript. All authors contributed to the article and approved the submitted version. 

## Funding

This work was supported by the Science and Technology Commission of Shanghai Municipality (19511104401), and the Young Doctor Foundation of Colleges and Universities in Gansu Province (2022QB-153) and the Natural Science Foundation of China (31670223 and 31270407).

## Conflict of interest

The authors declare that the research was conducted in the absence of any commercial or financial relationships that could be construed as a potential conflict of interest.

## Publisher’s note

All claims expressed in this article are solely those of the authors and do not necessarily represent those of their affiliated organizations, or those of the publisher, the editors and the reviewers. Any product that may be evaluated in this article, or claim that may be made by its manufacturer, is not guaranteed or endorsed by the publisher.
