# Supplementary material for: Population genetic structure of Wikstroemia monnula highlights the necessity and feasibility of hierarchical analysis for a highly differentiated species
Source: Front Plant Sci. 2022 Oct 7;13:962364. doi: 10.3389/fpls.2022.962364 (PMC9624186; doi:10.3389/fpls.2022.962364)
Supplement: Supplementary file 1 [file DataSheet_1.docx]

| **Tab. S1 Geographic information of the 38 sampled population of *Wikstroemia monnual*.** | | | | | |
| --- | --- | --- | --- | --- | --- |
| Population code | Locations | Longitude (°E) | Latitude(°N) | Altitude(m) | N |
| P2 | Mt.Sanqing, Zhejiang | 118.08 | 28.88 | 895-925 | 30 |
| P3 | Mt.Gutian, Zhejiang | 118.11 | 29.26 | 756-810 | 21 |
| P6 | Mt. Jiulong, Zhejiang | 118.98 | 28.34 | 800-1064 | 24 |
| P7 | Wangshukouzhen, Zhejiang | 119.05 | 28.44 | 470-480 | 24 |
| P8 | Menggaojiedao, Zhejiang | 119.4 | 28.66 | 359-460 | 24 |
| P10 | Shangyuzhen, Zhejiang | 118.76 | 28.73 | 581-610 | 20 |
| P11 | Mt. Jinggang1, Jiangxi | 114.16 | 26.51 | 1340-1348 | 30 |
| P12 | Mt. Jinggang2, Jiangxi | 114.18 | 26.56 | 760-780 | 20 |
| P13 | Mt. Dayao, Guangdong | 113.21 | 25.36 | 800-860 | 25 |
| P14 | Mt. Mang, Hunan | 112.93 | 24.98 | 966-1060 | 33 |
| P15 | Nanling, Guangdong | 113.06 | 24.9 | 920-960 | 33 |
| P16 | Mt. Nankun,Guangdong | 113.82 | 23.66 | 1172-1230 | 30 |
| P17 | Huaping, Guangxi | 109.88 | 25.59 | 1230-1300 | 31 |
| P18 | Xiuning, Anhui | 118.27 | 29.42 | 750-800 | 21 |
| P19 | Mt. Liangye1, Fujian | 116.28 | 25.28 | 1140-1200 | 23 |
| P20 | Mt. Liangye2, Fujian | 116.28 | 25.17 | 880-900 | 21 |
| P21 | Mt. Mangdang, Fujian | 118.11 | 26.71 | 1002-1130 | 28 |
| P22 | Chenhedong, Guangdong | 113.91 | 23.75 | 870-880 | 26 |
| P25 | Mt. Wuyi, Fujian | 117.93 | 27.67 | 200-301 | 24 |
| P26 | Taining, Fujian | 117.19 | 26.98 | 491-549 | 24 |
| P27 | Mt. Wu, Fujian | 117.18 | 23.72 | 861-876 | 24 |
| P28 | Mt. Qiniang, Guangdong | 114.55 | 22.53 | 780-812 | 24 |
| P29 | Mt. Dinghu, Guangdong | 112.54 | 23.17 | 750-865 | 24 |
| P30 | Mt. Hewei, Guangdong | 111.42 | 21.84 | 936-1085 | 24 |
| P31 | Mt. Yuanbao, Guangxi | 109.16 | 25.42 | 1287-1365 | 24 |
| P32 | Mt. Dayao, Guangxi | 110.32 | 24.22 | 791-1361 | 24 |
| P33 | Wenzhou, Zhejiang | 120.42 | 28.38 | 789-799 | 18 |
| P34 | Mt. Yandang, Zhejiang | 121.09 | 28.38 | 721-731 | 24 |
| P35 | Wuzhifeng, Jiangxi | 114.53 | 25.8 | 793-799 | 24 |
| P36 | Mt. Zuobayun, Jiangxi | 114.24 | 26.22 | 1056-1139 | 24 |
| P37 | Mt. Xiang, Jiangxi | 115.65 | 24.95 | 993-1158 | 20 |
| P38 | Mt. Bamian, Jiangxi | 114.09 | 26.39 | 855-1096 | 24 |
| P39 | Mt.Wugong, Jiangxi | 114.06 | 26.29 | 810-1038 | 11 |
| P40 | Hechengqu, Hunan | 110.26 | 27.72 | 822-854 | 24 |
| P41 | Chengbuxian, Hunan | 110.12 | 27.55 | 729-773 | 24 |
| P42 | Mt. Wugangyun, Hunan | 110.61 | 26.41 | 659-720 | 9 |
| P43 | Mt. Gupo, Hunan | 110.61 | 26.66 | 686-933 | 23 |
| P44 | Leanxian, Jiangxi | 115.91 | 27.11 | 798-803 | 24 |
| Species mean |  |  |  |  | 23.82 |
| Species total |  |  | | | 905 |
| N, sample size for nSSR analysis. | | | | | |

| **Tab. S2 The informations of twelve EST-SSR markers in this study.** | | | | | | | |  |
| --- | --- | --- | --- | --- | --- | --- | --- | --- |
| Locus | Primer (5‘→3′) | Repeat motif | Ta (℃) | | Size | | |  |
| RH1 | F-TCAAACCCAGTGGAGTGATCAGGAC | (ATC)7 | 58.5 | | 128-149 | | |  |
|  | R-GCAAGAACAAGTTCATGCGAAAAGA |  |  | |  | | |  |
| RH2 | F-GCTATGCCAGCCTACAGGTGGAAAG | (GTG)7 | 61 | | 273-294 | | |  |
|  | R-GCTATGCCAGCCTACAGGTGGAAAG |  |  | |  | | |  |
| RH3 | F-CGACGCTGATGAAGTTGACTCTGAC | (CGA)8 | 60 | | 230-251 | | |  |
|  | R-GAGTCCTGGAAGGCAGAATGTTGTC |  |  | |  | | |  |
| RH4 | F-GGAGAATGATGATTGGAGGAAGAAG | (AATCC)5 | 58 | | 192-217 | | |  |
|  | R-CAAATCAACTAACCATCCTTGTGTG |  |  | |  | | |  |
| RH5 | F-GGCACTGATTCTGAGTTTCCATTGT | (CTTGTC)5 | 59 | | 398-428 | | |  |
|  | R-GAGTTGTATGGAGATGCTGGGGTTC |  |  | |  | | |  |
| RH6 | F-GAAATGGACCCAAGAATGCC | (CCTCCA)5 | 56 | | 393-423 | | |  |
|  | R-AACTCTTGAATCACTACCTCTCGCT |  |  | |  | | |  |
| RH7 | F-TAAAACCCTACTCCTGGTGGTCTAT | (CTG)7 | 56.5 | | 126-147 | | |  |
|  | R-TTCTCAACCTCAACAACAGACTCAG |  |  | |  | | |  |
| RH8 | F-ACCTTGCAGATGCTATTGTTGATTC | (TC)10 | 56 | | 240-260 | | |  |
|  | R-ACCTTGCAGATGCTATTGTTGATTC |  |  | |  | | |  |
| RH9 | F-GAGAGAGGTTTGCGGGAAAGGGAAG | (ATGC)6 | 62 | | 232-253 | | |  |
|  | R-CGTTGAACATACGACGGAGAGCGAC |  |  | |  | | |  |
| RH10 | F-TTGGGAAAAGAGTGAAGTGAAGGTC | (TC)10 | 56 | | 250-270 | | |  |
|  | R-AGTAACTGAAAAAAGAGGGGCAATG |  | | | |  |  | |
| RH11 | F-TTATGCCCGACAAGAAGAGTAGAAG | (CCA)7 | 56 | | 218-239 | | |  |
|  | R-CTCTTCCTTCATCTCTCACCGTTAG |  | |  |  | | |  |
| RH12 | F-AGAGATTCGTGCTACAGAGCCTTTG | (TGA)7 | 57.5 | | 186-207 | | |  |
|  | R-TCTTCATCGCCATCACTATTGTCAC |  | |  |  | | |  |

| **Tab. S3 Gene flow（*N*m）in 38 population of *Wikstroemia monnula.*** | | | | | | | | | | | | | | | | | | | | |
| --- | --- | --- | --- | --- | --- | --- | --- | --- | --- | --- | --- | --- | --- | --- | --- | --- | --- | --- | --- | --- |
|  | P2 | P3 | P6 | P7 | P8 | P10 | P11 | P12 | P13 | P14 | P15 | P16 | P17 | P18 | P19 | P20 | P21 | P22 | P25 | P26 |
| P2 | 0 |  |  |  |  |  |  |  |  |  |  |  |  |  |  |  |  |  |  |  |
| P3 | 0.038 | 0 |  |  |  |  |  |  |  |  |  |  |  |  |  |  |  |  |  |  |
| P6 | 0.044 | 0.028 | 0 |  |  |  |  |  |  |  |  |  |  |  |  |  |  |  |  |  |
| P7 | 0.051 | 0.023 | 0.026 | 0 |  |  |  |  |  |  |  |  |  |  |  |  |  |  |  |  |
| P8 | 0.055 | 0.037 | 0.048 | 0.04 | 0 |  |  |  |  |  |  |  |  |  |  |  |  |  |  |  |
| P10 | 0.044 | 0.046 | 0.038 | 0.053 | 0.056 | 0 |  |  |  |  |  |  |  |  |  |  |  |  |  |  |
| P11 | 0.048 | 0.051 | 0.045 | 0.057 | 0.058 | 0.005 | 0 |  |  |  |  |  |  |  |  |  |  |  |  |  |
| P12 | 0.052 | 0.028 | 0.04 | 0.035 | 0.02 | 0.05 | 0.054 | 0 |  |  |  |  |  |  |  |  |  |  |  |  |
| P13 | 0.058 | 0.061 | 0.062 | 0.062 | 0.062 | 0.059 | 0.059 | 0.061 | 0 |  |  |  |  |  |  |  |  |  |  |  |
| P14 | 0.06 | 0.057 | 0.06 | 0.061 | 0.062 | 0.061 | 0.062 | 0.058 | 0.062 | 0 |  |  |  |  |  |  |  |  |  |  |
| P15 | 0.051 | 0.046 | 0.056 | 0.056 | 0.053 | 0.051 | 0.056 | 0.049 | 0.06 | 0.059 | 0 |  |  |  |  |  |  |  |  |  |
| P16 | 0.061 | 0.06 | 0.062 | 0.063 | 0.063 | 0.062 | 0.06 | 0.062 | 0.051 | 0.058 | 0.063 | 0 |  |  |  |  |  |  |  |  |
| P17 | 0.06 | 0.055 | 0.061 | 0.058 | 0.06 | 0.063 | 0.062 | 0.057 | 0.063 | 0.062 | 0.06 | 0.062 | 0 |  |  |  |  |  |  |  |
| P18 | 0.062 | 0.056 | 0.061 | 0.058 | 0.062 | 0.062 | 0.061 | 0.058 | 0.059 | 0.062 | 0.062 | 0.06 | 0.063 | 0 |  |  |  |  |  |  |
| P19 | 0.06 | 0.054 | 0.061 | 0.061 | 0.056 | 0.062 | 0.063 | 0.055 | 0.062 | 0.063 | 0.06 | 0.06 | 0.062 | 0.062 | 0 |  |  |  |  |  |
| P20 | 0.051 | 0.04 | 0.048 | 0.05 | 0.051 | 0.052 | 0.056 | 0.038 | 0.061 | 0.053 | 0.048 | 0.057 | 0.051 | 0.053 | 0.058 | 0 |  |  |  |  |
| P21 | 0.056 | 0.048 | 0.053 | 0.055 | 0.052 | 0.058 | 0.06 | 0.043 | 0.063 | 0.061 | 0.052 | 0.061 | 0.062 | 0.062 | 0.061 | 0.033 | 0 |  |  |  |
| P22 | 0.057 | 0.055 | 0.045 | 0.057 | 0.06 | 0.03 | 0.034 | 0.058 | 0.062 | 0.062 | 0.062 | 0.052 | 0.058 | 0.056 | 0.06 | 0.06 | 0.062 | 0 |  |  |
| P25 | 0.056 | 0.038 | 0.052 | 0.05 | 0.057 | 0.059 | 0.061 | 0.051 | 0.062 | 0.059 | 0.06 | 0.061 | 0.063 | 0.061 | 0.062 | 0.051 | 0.058 | 0.062 | 0 |  |
| P26 | 0.054 | 0.04 | 0.049 | 0.05 | 0.056 | 0.054 | 0.057 | 0.046 | 0.063 | 0.057 | 0.057 | 0.062 | 0.062 | 0.062 | 0.062 | 0.049 | 0.056 | 0.061 | 0.046 | 0 |
| P27 | 0.052 | 0.031 | 0.051 | 0.05 | 0.054 | 0.053 | 0.058 | 0.045 | 0.063 | 0.057 | 0.054 | 0.061 | 0.062 | 0.061 | 0.06 | 0.042 | 0.053 | 0.062 | 0.039 | 0.044 |
| P28 | 0.053 | 0.041 | 0.056 | 0.056 | 0.054 | 0.057 | 0.059 | 0.05 | 0.063 | 0.062 | 0.058 | 0.062 | 0.063 | 0.063 | 0.056 | 0.051 | 0.057 | 0.062 | 0.057 | 0.056 |
| P29 | 0.049 | 0.047 | 0.052 | 0.053 | 0.05 | 0.051 | 0.055 | 0.045 | 0.061 | 0.058 | 0.05 | 0.062 | 0.062 | 0.062 | 0.057 | 0.048 | 0.051 | 0.061 | 0.053 | 0.049 |
| P30 | 0.057 | 0.058 | 0.06 | 0.062 | 0.061 | 0.06 | 0.061 | 0.058 | 0.063 | 0.06 | 0.06 | 0.06 | 0.062 | 0.063 | 0.063 | 0.055 | 0.061 | 0.063 | 0.062 | 0.061 |
| **Tab. S3** (continued) | | | | | | | | | | | | | | | | | | | | |
|  | P2 | P3 | P6 | P7 | P8 | P10 | P11 | P12 | P13 | P14 | P15 | P16 | P17 | P18 | P19 | P20 | P21 | P22 | P25 | P26 |
| P31 | 0.057 | 0.058 | 0.059 | 0.061 | 0.06 | 0.058 | 0.06 | 0.057 | 0.062 | 0.063 | 0.059 | 0.06 | 0.061 | 0.061 | 0.062 | 0.059 | 0.062 | 0.063 | 0.062 | 0.062 |
| P32 | 0.05 | 0.054 | 0.056 | 0.06 | 0.057 | 0.057 | 0.059 | 0.055 | 0.062 | 0.062 | 0.056 | 0.062 | 0.063 | 0.062 | 0.06 | 0.056 | 0.059 | 0.063 | 0.061 | 0.061 |
| P33 | 0.053 | 0.057 | 0.06 | 0.062 | 0.059 | 0.059 | 0.061 | 0.058 | 0.062 | 0.062 | 0.057 | 0.06 | 0.062 | 0.062 | 0.061 | 0.059 | 0.061 | 0.062 | 0.062 | 0.062 |
| P34 | 0.046 | 0.052 | 0.053 | 0.058 | 0.058 | 0.054 | 0.057 | 0.054 | 0.063 | 0.062 | 0.055 | 0.062 | 0.063 | 0.063 | 0.061 | 0.055 | 0.059 | 0.062 | 0.06 | 0.06 |
| P35 | 0.062 | 0.06 | 0.061 | 0.062 | 0.062 | 0.062 | 0.062 | 0.059 | 0.059 | 0.062 | 0.063 | 0.055 | 0.06 | 0.062 | 0.062 | 0.059 | 0.062 | 0.06 | 0.063 | 0.062 |
| P36 | 0.054 | 0.053 | 0.052 | 0.058 | 0.061 | 0.057 | 0.059 | 0.052 | 0.062 | 0.062 | 0.06 | 0.061 | 0.062 | 0.063 | 0.062 | 0.055 | 0.06 | 0.063 | 0.057 | 0.054 |
| P37 | 0.06 | 0.055 | 0.057 | 0.06 | 0.056 | 0.061 | 0.062 | 0.052 | 0.061 | 0.061 | 0.06 | 0.061 | 0.062 | 0.063 | 0.062 | 0.056 | 0.061 | 0.062 | 0.061 | 0.059 |
| P38 | 0.052 | 0.057 | 0.058 | 0.061 | 0.061 | 0.058 | 0.06 | 0.057 | 0.062 | 0.062 | 0.059 | 0.061 | 0.062 | 0.062 | 0.063 | 0.059 | 0.062 | 0.062 | 0.062 | 0.062 |
| P39 | 0.055 | 0.055 | 0.055 | 0.06 | 0.059 | 0.057 | 0.06 | 0.055 | 0.062 | 0.062 | 0.057 | 0.062 | 0.062 | 0.062 | 0.06 | 0.055 | 0.058 | 0.063 | 0.062 | 0.061 |
| P40 | 0.059 | 0.054 | 0.058 | 0.06 | 0.059 | 0.061 | 0.062 | 0.055 | 0.062 | 0.06 | 0.059 | 0.061 | 0.061 | 0.062 | 0.062 | 0.052 | 0.06 | 0.062 | 0.062 | 0.061 |
| P41 | 0.061 | 0.062 | 0.062 | 0.061 | 0.06 | 0.059 | 0.057 | 0.062 | 0.05 | 0.061 | 0.062 | 0.04 | 0.056 | 0.057 | 0.054 | 0.063 | 0.059 | 0.045 | 0.06 | 0.06 |
| P42 | 0.062 | 0.062 | 0.061 | 0.06 | 0.062 | 0.059 | 0.057 | 0.063 | 0.051 | 0.06 | 0.062 | 0.049 | 0.057 | 0.052 | 0.054 | 0.062 | 0.062 | 0.049 | 0.058 | 0.059 |
| P43 | 0.061 | 0.061 | 0.061 | 0.062 | 0.063 | 0.062 | 0.063 | 0.06 | 0.056 | 0.062 | 0.063 | 0.056 | 0.059 | 0.06 | 0.061 | 0.059 | 0.062 | 0.059 | 0.062 | 0.062 |
| P44 | 0.062 | 0.061 | 0.062 | 0.062 | 0.063 | 0.063 | 0.062 | 0.058 | 0.054 | 0.062 | 0.063 | 0.048 | 0.057 | 0.06 | 0.059 | 0.061 | 0.062 | 0.056 | 0.063 | 0.063 |

| **Tab. S3** (Continued) | | | | | | | | | | | | | | | | | | |
| --- | --- | --- | --- | --- | --- | --- | --- | --- | --- | --- | --- | --- | --- | --- | --- | --- | --- | --- |
|  | P27 | P28 | P29 | P30 | P31 | P32 | P33 | P34 | P35 | P36 | P37 | P38 | P39 | P40 | P41 | P42 | P43 | P44 |
| P27 | 0 |  |  |  |  |  |  |  |  |  |  |  |  |  |  |  |  |  |
| P28 | 0.048 | 0 |  |  |  |  |  |  |  |  |  |  |  |  |  |  |  |  |
| P29 | 0.053 | 0.054 | 0 |  |  |  |  |  |  |  |  |  |  |  |  |  |  |  |
| P30 | 0.061 | 0.061 | 0.059 | 0 |  |  |  |  |  |  |  |  |  |  |  |  |  |  |
| P31 | 0.062 | 0.059 | 0.052 | 0.061 | 0 |  |  |  |  |  |  |  |  |  |  |  |  |  |
| P32 | 0.059 | 0.054 | 0.05 | 0.059 | 0.039 | 0 |  |  |  |  |  |  |  |  |  |  |  |  |
| P33 | 0.061 | 0.059 | 0.052 | 0.062 | 0.044 | 0.021 | 0 |  |  |  |  |  |  |  |  |  |  |  |
| P34 | 0.059 | 0.057 | 0.052 | 0.059 | 0.031 | 0.029 | 0.03 | 0 |  |  |  |  |  |  |  |  |  |  |
| P35 | 0.062 | 0.061 | 0.06 | 0.056 | 0.062 | 0.061 | 0.062 | 0.061 | 0 |  |  |  |  |  |  |  |  |  |
| P36 | 0.057 | 0.057 | 0.054 | 0.061 | 0.057 | 0.056 | 0.06 | 0.053 | 0.062 | 0 |  |  |  |  |  |  |  |  |
| P37 | 0.061 | 0.057 | 0.052 | 0.055 | 0.058 | 0.056 | 0.06 | 0.058 | 0.057 | 0.06 | 0 |  |  |  |  |  |  |  |
| P38 | 0.062 | 0.061 | 0.053 | 0.061 | 0.021 | 0.033 | 0.037 | 0.029 | 0.062 | 0.057 | 0.059 | 0 |  |  |  |  |  |  |
| P39 | 0.06 | 0.055 | 0.053 | 0.061 | 0.048 | 0.022 | 0.045 | 0.039 | 0.062 | 0.058 | 0.059 | 0.047 | 0 |  |  |  |  |  |
| P40 | 0.06 | 0.056 | 0.055 | 0.055 | 0.06 | 0.056 | 0.06 | 0.058 | 0.055 | 0.06 | 0.047 | 0.061 | 0.058 | 0 |  |  |  |  |
| P41 | 0.053 | 0.057 | 0.062 | 0.063 | 0.058 | 0.059 | 0.057 | 0.06 | 0.06 | 0.059 | 0.062 | 0.057 | 0.057 | 0.062 | 0 |  |  |  |
| P42 | 0.054 | 0.058 | 0.062 | 0.062 | 0.058 | 0.06 | 0.058 | 0.06 | 0.059 | 0.057 | 0.062 | 0.058 | 0.059 | 0.062 | 0.057 | 0 |  |  |
| P43 | 0.062 | 0.061 | 0.062 | 0.062 | 0.062 | 0.059 | 0.062 | 0.055 | 0.061 | 0.061 | 0.061 | 0.062 | 0.058 | 0.061 | 0.053 | 0.054 | 0 |  |
| P44 | 0.062 | 0.063 | 0.062 | 0.062 | 0.062 | 0.062 | 0.063 | 0.059 | 0.063 | 0.055 | 0.062 | 0.062 | 0.062 | 0.063 | 0.048 | 0.048 | 0.06 | 0 |

| **Tab. S4 Population genetic differentiation（*F*_ST_）among 38 population of *Wikstroemia monnula*.** | | | | | | | | | | | | | | | | | | | | |
| --- | --- | --- | --- | --- | --- | --- | --- | --- | --- | --- | --- | --- | --- | --- | --- | --- | --- | --- | --- | --- |
|  | P2 | P3 | P6 | P7 | P8 | P10 | P11 | P12 | P13 | P14 | P15 | P16 | P17 | P18 | P19 | P20 | P21 | P22 | P25 | P26 |
| P2 | 0.000 |  |  |  |  |  |  |  |  |  |  |  |  |  |  |  |  |  |  |  |
| P3 | 0.189 | 0.000 |  |  |  |  |  |  |  |  |  |  |  |  |  |  |  |  |  |  |
| P6 | 0.229 | 0.129 | 0.000 |  |  |  |  |  |  |  |  |  |  |  |  |  |  |  |  |  |
| P7 | 0.289 | 0.103 | 0.120 | 0.000 |  |  |  |  |  |  |  |  |  |  |  |  |  |  |  |  |
| P8 | 0.331 | 0.180 | 0.262 | 0.203 | 0.000 |  |  |  |  |  |  |  |  |  |  |  |  |  |  |  |
| P10 | 0.229 | 0.241 | 0.184 | 0.303 | 0.341 | 0.000 |  |  |  |  |  |  |  |  |  |  |  |  |  |  |
| P11 | 0.258 | 0.284 | 0.234 | 0.349 | 0.369 | 0.022 | 0.000 |  |  |  |  |  |  |  |  |  |  |  |  |  |
| P12 | 0.291 | 0.127 | 0.202 | 0.168 | 0.086 | 0.280 | 0.316 | 0.000 |  |  |  |  |  |  |  |  |  |  |  |  |
| P13 | 0.369 | 0.411 | 0.477 | 0.478 | 0.463 | 0.374 | 0.380 | 0.416 | 0.000 |  |  |  |  |  |  |  |  |  |  |  |
| P14 | 0.401 | 0.351 | 0.408 | 0.422 | 0.442 | 0.421 | 0.468 | 0.368 | 0.559 | 0.000 |  |  |  |  |  |  |  |  |  |  |
| P15 | 0.284 | 0.241 | 0.339 | 0.341 | 0.307 | 0.290 | 0.343 | 0.263 | 0.399 | 0.376 | 0.000 |  |  |  |  |  |  |  |  |  |
| P16 | 0.422 | 0.401 | 0.454 | 0.486 | 0.492 | 0.552 | 0.607 | 0.438 | 0.711 | 0.637 | 0.499 | 0.000 |  |  |  |  |  |  |  |  |
| P17 | 0.404 | 0.327 | 0.425 | 0.372 | 0.409 | 0.505 | 0.529 | 0.353 | 0.502 | 0.524 | 0.404 | 0.562 | 0.000 |  |  |  |  |  |  |  |
| P18 | 0.446 | 0.333 | 0.425 | 0.370 | 0.474 | 0.542 | 0.588 | 0.371 | 0.619 | 0.480 | 0.450 | 0.610 | 0.511 | 0.000 |  |  |  |  |  |  |
| P19 | 0.403 | 0.311 | 0.431 | 0.414 | 0.341 | 0.470 | 0.488 | 0.332 | 0.536 | 0.508 | 0.406 | 0.595 | 0.525 | 0.526 | 0.000 |  |  |  |  |  |
| P20 | 0.287 | 0.197 | 0.258 | 0.272 | 0.283 | 0.297 | 0.333 | 0.185 | 0.434 | 0.304 | 0.263 | 0.353 | 0.286 | 0.302 | 0.363 | 0.000 |  |  |  |  |
| P21 | 0.337 | 0.260 | 0.301 | 0.328 | 0.297 | 0.358 | 0.392 | 0.224 | 0.513 | 0.410 | 0.299 | 0.422 | 0.441 | 0.467 | 0.431 | 0.156 | 0.000 |  |  |  |
| P22 | 0.358 | 0.327 | 0.233 | 0.352 | 0.403 | 0.139 | 0.159 | 0.359 | 0.532 | 0.550 | 0.452 | 0.706 | 0.629 | 0.662 | 0.601 | 0.407 | 0.470 | 0.000 |  |  |
| P25 | 0.336 | 0.187 | 0.298 | 0.278 | 0.353 | 0.379 | 0.430 | 0.281 | 0.556 | 0.387 | 0.393 | 0.568 | 0.494 | 0.435 | 0.437 | 0.282 | 0.367 | 0.485 | 0.000 |  |
| P26 | 0.313 | 0.200 | 0.270 | 0.280 | 0.338 | 0.311 | 0.357 | 0.245 | 0.508 | 0.348 | 0.345 | 0.546 | 0.483 | 0.446 | 0.441 | 0.268 | 0.333 | 0.434 | 0.241 | 0.000 |
| P27 | 0.298 | 0.148 | 0.285 | 0.279 | 0.318 | 0.307 | 0.370 | 0.238 | 0.490 | 0.354 | 0.319 | 0.581 | 0.465 | 0.432 | 0.397 | 0.212 | 0.304 | 0.471 | 0.193 | 0.227 |
| P28 | 0.308 | 0.209 | 0.333 | 0.340 | 0.310 | 0.348 | 0.379 | 0.276 | 0.495 | 0.453 | 0.364 | 0.516 | 0.492 | 0.488 | 0.337 | 0.284 | 0.349 | 0.485 | 0.357 | 0.340 |
| **Tab. S4** (continued) | | | | | | | | | | | | | | | | | | | | |
|  | P2 | P3 | P6 | P7 | P8 | P10 | P11 | P12 | P13 | P14 | P15 | P16 | P17 | P18 | P19 | P20 | P21 | P22 | P25 | P26 |
| P29 | 0.263 | 0.249 | 0.292 | 0.309 | 0.274 | 0.290 | 0.321 | 0.237 | 0.427 | 0.363 | 0.280 | 0.521 | 0.447 | 0.452 | 0.357 | 0.258 | 0.289 | 0.424 | 0.306 | 0.267 |
| P30 | 0.349 | 0.359 | 0.400 | 0.440 | 0.435 | 0.397 | 0.414 | 0.370 | 0.505 | 0.402 | 0.399 | 0.592 | 0.519 | 0.492 | 0.509 | 0.331 | 0.427 | 0.505 | 0.453 | 0.416 |
| P31 | 0.345 | 0.362 | 0.376 | 0.432 | 0.396 | 0.366 | 0.404 | 0.347 | 0.550 | 0.512 | 0.374 | 0.609 | 0.567 | 0.569 | 0.464 | 0.385 | 0.440 | 0.513 | 0.440 | 0.446 |
| P32 | 0.274 | 0.314 | 0.336 | 0.393 | 0.357 | 0.351 | 0.386 | 0.321 | 0.519 | 0.449 | 0.334 | 0.543 | 0.508 | 0.526 | 0.402 | 0.337 | 0.386 | 0.486 | 0.434 | 0.425 |
| P33 | 0.302 | 0.352 | 0.394 | 0.435 | 0.383 | 0.383 | 0.410 | 0.367 | 0.528 | 0.477 | 0.353 | 0.603 | 0.551 | 0.556 | 0.419 | 0.387 | 0.430 | 0.539 | 0.470 | 0.461 |
| P34 | 0.244 | 0.292 | 0.309 | 0.363 | 0.358 | 0.315 | 0.345 | 0.313 | 0.495 | 0.454 | 0.322 | 0.543 | 0.493 | 0.513 | 0.420 | 0.330 | 0.377 | 0.459 | 0.407 | 0.404 |
| P35 | 0.437 | 0.409 | 0.425 | 0.457 | 0.461 | 0.485 | 0.515 | 0.384 | 0.627 | 0.459 | 0.490 | 0.677 | 0.599 | 0.562 | 0.554 | 0.389 | 0.469 | 0.606 | 0.506 | 0.459 |
| P36 | 0.312 | 0.300 | 0.298 | 0.369 | 0.418 | 0.345 | 0.379 | 0.297 | 0.553 | 0.449 | 0.409 | 0.581 | 0.540 | 0.510 | 0.476 | 0.331 | 0.405 | 0.504 | 0.353 | 0.320 |
| P37 | 0.394 | 0.329 | 0.351 | 0.393 | 0.339 | 0.411 | 0.446 | 0.299 | 0.567 | 0.427 | 0.404 | 0.588 | 0.530 | 0.500 | 0.459 | 0.335 | 0.425 | 0.528 | 0.432 | 0.375 |
| P38 | 0.299 | 0.354 | 0.366 | 0.434 | 0.416 | 0.370 | 0.407 | 0.353 | 0.557 | 0.517 | 0.373 | 0.579 | 0.554 | 0.563 | 0.486 | 0.381 | 0.439 | 0.524 | 0.471 | 0.440 |
| P39 | 0.321 | 0.321 | 0.325 | 0.396 | 0.377 | 0.355 | 0.399 | 0.326 | 0.526 | 0.458 | 0.352 | 0.535 | 0.519 | 0.525 | 0.409 | 0.332 | 0.366 | 0.488 | 0.446 | 0.427 |
| P40 | 0.373 | 0.317 | 0.368 | 0.390 | 0.385 | 0.423 | 0.449 | 0.322 | 0.525 | 0.401 | 0.373 | 0.579 | 0.429 | 0.454 | 0.442 | 0.290 | 0.406 | 0.551 | 0.450 | 0.413 |
| P41 | 0.569 | 0.539 | 0.558 | 0.566 | 0.600 | 0.618 | 0.653 | 0.541 | 0.722 | 0.585 | 0.559 | 0.803 | 0.667 | 0.654 | 0.688 | 0.506 | 0.626 | 0.763 | 0.605 | 0.591 |
| P42 | 0.557 | 0.543 | 0.580 | 0.607 | 0.561 | 0.616 | 0.644 | 0.511 | 0.714 | 0.602 | 0.550 | 0.732 | 0.643 | 0.709 | 0.681 | 0.472 | 0.557 | 0.731 | 0.640 | 0.627 |
| P43 | 0.429 | 0.428 | 0.435 | 0.478 | 0.504 | 0.459 | 0.510 | 0.404 | 0.664 | 0.473 | 0.489 | 0.659 | 0.616 | 0.604 | 0.575 | 0.373 | 0.465 | 0.624 | 0.516 | 0.480 |
| P44 | 0.460 | 0.418 | 0.448 | 0.481 | 0.505 | 0.501 | 0.558 | 0.370 | 0.683 | 0.525 | 0.487 | 0.743 | 0.649 | 0.603 | 0.616 | 0.429 | 0.530 | 0.668 | 0.507 | 0.486 |

| **Tab. S4** (continued) | | | | | | | | | | | | | | | | | | |
| --- | --- | --- | --- | --- | --- | --- | --- | --- | --- | --- | --- | --- | --- | --- | --- | --- | --- | --- |
|  | P27 | P28 | P29 | P30 | P31 | P32 | P33 | P34 | P35 | P36 | P37 | P38 | P39 | P40 | P41 | P42 | P43 | P44 |
| P27 | 0.000 |  |  |  |  |  |  |  |  |  |  |  |  |  |  |  |  |  |
| P28 | 0.258 | 0.000 |  |  |  |  |  |  |  |  |  |  |  |  |  |  |  |  |
| P29 | 0.302 | 0.313 | 0.000 |  |  |  |  |  |  |  |  |  |  |  |  |  |  |  |
| P30 | 0.433 | 0.413 | 0.376 | 0.000 |  |  |  |  |  |  |  |  |  |  |  |  |  |  |
| P31 | 0.439 | 0.389 | 0.291 | 0.434 | 0.000 |  |  |  |  |  |  |  |  |  |  |  |  |  |
| P32 | 0.387 | 0.310 | 0.280 | 0.383 | 0.195 | 0.000 |  |  |  |  |  |  |  |  |  |  |  |  |
| P33 | 0.420 | 0.382 | 0.296 | 0.440 | 0.230 | 0.093 | 0.000 |  |  |  |  |  |  |  |  |  |  |  |
| P34 | 0.377 | 0.346 | 0.292 | 0.377 | 0.146 | 0.135 | 0.141 | 0.000 |  |  |  |  |  |  |  |  |  |  |
| P35 | 0.529 | 0.435 | 0.390 | 0.338 | 0.467 | 0.413 | 0.480 | 0.410 | 0.000 |  |  |  |  |  |  |  |  |  |
| P36 | 0.355 | 0.350 | 0.320 | 0.418 | 0.352 | 0.340 | 0.405 | 0.305 | 0.458 | 0.000 |  |  |  |  |  |  |  |  |
| P37 | 0.418 | 0.355 | 0.292 | 0.325 | 0.372 | 0.339 | 0.400 | 0.367 | 0.348 | 0.395 | 0.000 |  |  |  |  |  |  |  |
| P38 | 0.451 | 0.411 | 0.301 | 0.415 | 0.094 | 0.156 | 0.179 | 0.131 | 0.468 | 0.357 | 0.376 | 0.000 |  |  |  |  |  |  |
| P39 | 0.399 | 0.326 | 0.309 | 0.435 | 0.262 | 0.099 | 0.233 | 0.191 | 0.450 | 0.358 | 0.389 | 0.255 | 0.000 |  |  |  |  |  |
| P40 | 0.395 | 0.335 | 0.322 | 0.328 | 0.402 | 0.342 | 0.406 | 0.364 | 0.323 | 0.409 | 0.248 | 0.419 | 0.370 | 0.000 |  |  |  |  |
| P41 | 0.695 | 0.651 | 0.562 | 0.499 | 0.637 | 0.624 | 0.652 | 0.608 | 0.592 | 0.611 | 0.559 | 0.652 | 0.642 | 0.537 | 0.000 |  |  |  |
| P42 | 0.680 | 0.632 | 0.564 | 0.484 | 0.641 | 0.595 | 0.640 | 0.601 | 0.621 | 0.644 | 0.564 | 0.637 | 0.619 | 0.541 | 0.647 | 0.000 |  |  |
| P43 | 0.527 | 0.430 | 0.439 | 0.477 | 0.442 | 0.376 | 0.454 | 0.328 | 0.410 | 0.410 | 0.426 | 0.453 | 0.369 | 0.434 | 0.698 | 0.689 | 0.000 |  |
| P44 | 0.533 | 0.508 | 0.452 | 0.477 | 0.451 | 0.438 | 0.514 | 0.382 | 0.486 | 0.327 | 0.446 | 0.457 | 0.481 | 0.486 | 0.743 | 0.740 | 0.404 | 0.000 |

**Tab. S5 The analysis of molecular variance (AMOVA) of *Wikstroemia monnual*.**

| Source of variation | df | SS | Var.components | Pencentage of var. | *P*-value |
| --- | --- | --- | --- | --- | --- |
| Among groups | 3 | 1007 | 0.58 | 12.66 | 0.0001 |
| Among populations | 35 | 2487.5 | 1.45 | 31.72 | 0.0001 |
| within groups |  |  |  |  |  |
| Within populations | 1811 | 4602.67 | 2.54 | 55.61 | 0.0001 |

| **Tab. S6 Geographic distance among 38 population of *Wikstroemia monnula*.** | | | | | | | | | | | | | | | | | | | | |
| --- | --- | --- | --- | --- | --- | --- | --- | --- | --- | --- | --- | --- | --- | --- | --- | --- | --- | --- | --- | --- |
|  | P2 | P3 | P6 | P7 | P8 | P10 | P11 | P12 | P13 | P14 | P15 | P16 | P17 | P18 | P19 | P20 | P21 | P22 | P25 | P26 |
| P2 | 0.0 |  |  |  |  |  |  |  |  |  |  |  |  |  |  |  |  |  |  |  |
| P3 | 41.2 | 0.0 |  |  |  |  |  |  |  |  |  |  |  |  |  |  |  |  |  |  |
| P6 | 97.8 | 125.7 | 0.0 |  |  |  |  |  |  |  |  |  |  |  |  |  |  |  |  |  |
| P7 | 107.0 | 130.9 | 15.7 | 0.0 |  |  |  |  |  |  |  |  |  |  |  |  |  |  |  |  |
| P8 | 119.8 | 135.3 | 45.1 | 30.1 | 0.0 |  |  |  |  |  |  |  |  |  |  |  |  |  |  |  |
| P10 | 66.9 | 84.2 | 46.3 | 47.6 | 53.2 | 0.0 |  |  |  |  |  |  |  |  |  |  |  |  |  |  |
| P11 | 467.5 | 492.8 | 509.2 | 524.9 | 553.4 | 516.6 | 0.0 |  |  |  |  |  |  |  |  |  |  |  |  |  |
| P12 | 463.5 | 488.6 | 506.0 | 521.7 | 550.1 | 513.0 | 5.6 | 0.0 |  |  |  |  |  |  |  |  |  |  |  |  |
| P13 | 620.1 | 647.7 | 652.8 | 668.3 | 697.7 | 664.9 | 158.7 | 163.8 | 0.0 |  |  |  |  |  |  |  |  |  |  |  |
| P14 | 671.3 | 699.4 | 701.6 | 717.0 | 746.6 | 715.0 | 211.5 | 216.6 | 52.8 | 0.0 |  |  |  |  |  |  |  |  |  |  |
| P15 | 664.5 | 693.3 | 692.8 | 708.2 | 737.9 | 707.1 | 208.8 | 214.0 | 52.3 | 16.5 | 0.0 |  |  |  |  |  |  |  |  |  |
| P16 | 719.4 | 753.7 | 726.9 | 741.3 | 771.3 | 750.1 | 318.9 | 324.5 | 198.2 | 171.3 | 157.9 | 0.0 |  |  |  |  |  |  |  |  |
| P17 | 899.4 | 917.7 | 953.0 | 968.6 | 996.1 | 955.0 | 448.4 | 450.7 | 342.9 | 318.7 | 334.9 | 454.2 | 0.0 |  |  |  |  |  |  |  |
| P18 | 64.8 | 26.8 | 134.6 | 136.9 | 135.3 | 89.5 | 518.8 | 514.5 | 674.0 | 725.8 | 719.8 | 780.4 | 941.8 | 0.0 |  |  |  |  |  |  |
| P19 | 104.7 | 128.8 | 14.4 | 2.3 | 30.9 | 45.5 | 523.5 | 520.3 | 667.1 | 715.9 | 707.1 | 740.6 | 967.1 | 135.0 | 0.0 |  |  |  |  |  |
| P20 | 112.3 | 135.9 | 19.9 | 5.4 | 28.4 | 52.3 | 528.6 | 525.5 | 671.5 | 720.1 | 711.2 | 743.2 | 972.7 | 141.5 | 7.6 | 0.0 |  |  |  |  |
| P21 | 242.5 | 283.7 | 202.5 | 214.1 | 242.6 | 239.3 | 384.3 | 383.0 | 501.9 | 545.1 | 533.6 | 541.3 | 830.0 | 305.5 | 214.2 | 214.5 | 0.0 |  |  |  |
| P22 | 706.3 | 740.6 | 713.4 | 727.8 | 757.9 | 736.8 | 309.0 | 314.7 | 193.2 | 169.6 | 155.5 | 13.5 | 459.3 | 767.4 | 727.2 | 729.8 | 527.7 | 0.0 |  |  |
| P25 | 133.1 | 174.3 | 118.7 | 133.8 | 163.7 | 142.7 | 396.2 | 393.4 | 535.8 | 584.0 | 574.9 | 609.1 | 843.1 | 197.2 | 132.8 | 136.5 | 110.4 | 595.7 | 0.0 |  |
| P26 | 226.6 | 265.6 | 224.1 | 238.9 | 269.0 | 247.8 | 306.6 | 304.6 | 436.6 | 483.2 | 473.3 | 503.1 | 754.8 | 291.1 | 238.1 | 241.2 | 87.9 | 489.7 | 106.0 | 0.0 |
| P27 | 557.7 | 598.6 | 519.9 | 530.2 | 557.1 | 558.3 | 419.3 | 422.4 | 432.0 | 447.7 | 431.4 | 345.1 | 766.3 | 622.0 | 530.6 | 529.9 | 319.1 | 334.2 | 424.8 | 341.4 |
| P28 | 788.0 | 825.6 | 778.2 | 791.2 | 820.6 | 808.2 | 443.2 | 448.7 | 341.5 | 317.1 | 303.6 | 145.7 | 584.3 | 851.8 | 791.0 | 792.3 | 579.6 | 148.6 | 665.6 | 562.5 |
| **Tab. S6** (continued) | | | | | | | | | | | | | | | | | | | | |
|  | P2 | P3 | P6 | P7 | P8 | P10 | P11 | P12 | P13 | P14 | P15 | P16 | P17 | P18 | P19 | P20 | P21 | P22 | P25 | P26 |
| P29 | 840.4 | 872.5 | 855.5 | 870.3 | 900.4 | 875.9 | 404.3 | 409.8 | 251.7 | 202.6 | 199.5 | 139.2 | 380.4 | 899.3 | 869.5 | 872.6 | 675.6 | 152.1 | 737.0 | 631.4 |
| P30 | 1027.7 | 1059.9 | 1041.2 | 1055.8 | 1085.9 | 1062.5 | 587.5 | 592.8 | 430.8 | 378.8 | 379.2 | 316.3 | 440.2 | 1086.6 | 1055.1 | 1058.0 | 857.6 | 329.8 | 922.9 | 817.1 |
| P31 | 961.6 | 979.1 | 1016.5 | 1032.2 | 1059.4 | 1017.9 | 513.1 | 515.3 | 406.9 | 380.8 | 396.7 | 508.9 | 65.0 | 1002.9 | 1030.6 | 1036.3 | 894.9 | 514.8 | 907.3 | 819.6 |
| P32 | 928.1 | 952.1 | 967.7 | 983.4 | 1012.4 | 977.1 | 460.8 | 464.6 | 317.6 | 274.5 | 287.2 | 359.3 | 152.7 | 977.7 | 982.1 | 986.8 | 818.9 | 367.8 | 851.9 | 754.0 |
| P33 | 279.9 | 296.1 | 187.0 | 174.4 | 161.3 | 214.3 | 667.8 | 665.5 | 798.0 | 842.9 | 832.0 | 837.8 | 1116.1 | 293.0 | 176.6 | 169.1 | 299.7 | 824.4 | 275.6 | 362.8 |
| P34 | 297.9 | 305.7 | 215.3 | 200.6 | 179.2 | 231.1 | 714.7 | 712.0 | 849.6 | 895.7 | 885.3 | 896.7 | 1162.0 | 297.1 | 202.5 | 195.7 | 356.3 | 883.2 | 318.8 | 413.0 |
| P35 | 291.8 | 322.7 | 323.1 | 338.7 | 367.7 | 334.0 | 187.6 | 184.9 | 330.9 | 381.0 | 373.5 | 434.0 | 635.3 | 349.5 | 337.4 | 342.2 | 209.2 | 421.2 | 208.6 | 124.0 |
| P36 | 480.3 | 507.8 | 515.4 | 531.0 | 560.1 | 525.9 | 37.2 | 42.3 | 139.9 | 191.7 | 186.4 | 285.1 | 452.5 | 534.2 | 529.8 | 534.4 | 377.5 | 274.8 | 399.8 | 304.6 |
| P37 | 519.5 | 550.2 | 543.6 | 558.9 | 588.7 | 559.3 | 106.6 | 111.9 | 117.8 | 160.4 | 149.9 | 219.8 | 457.5 | 577.0 | 557.9 | 561.8 | 385.1 | 208.7 | 425.4 | 323.3 |
| P38 | 383.9 | 409.5 | 427.3 | 443.0 | 471.2 | 433.4 | 83.6 | 79.7 | 239.5 | 291.9 | 287.5 | 379.4 | 526.9 | 435.6 | 441.6 | 446.9 | 315.8 | 368.1 | 316.2 | 233.0 |
| P39 | 488.5 | 514.6 | 527.4 | 543.1 | 571.9 | 536.2 | 25.0 | 30.6 | 134.8 | 187.5 | 184.2 | 294.7 | 434.8 | 540.8 | 541.8 | 546.7 | 395.5 | 285.1 | 413.0 | 320.4 |
| P40 | 797.8 | 807.7 | 867.9 | 883.0 | 907.3 | 860.1 | 413.0 | 412.5 | 384.5 | 390.2 | 405.7 | 558.4 | 213.5 | 828.8 | 881.2 | 887.7 | 786.4 | 558.4 | 771.4 | 701.8 |
| P41 | 797.9 | 807.2 | 869.1 | 884.1 | 908.1 | 860.6 | 418.8 | 418.2 | 394.1 | 400.8 | 416.2 | 569.5 | 225.2 | 828.0 | 882.2 | 888.8 | 790.4 | 569.4 | 773.6 | 705.3 |
| P42 | 784.5 | 800.3 | 843.9 | 859.4 | 885.9 | 842.6 | 352.5 | 353.7 | 285.6 | 281.3 | 297.5 | 444.3 | 128.7 | 823.6 | 857.8 | 863.7 | 736.6 | 445.6 | 738.3 | 656.7 |
| P43 | 773.5 | 788.2 | 835.4 | 850.9 | 876.9 | 832.7 | 351.5 | 352.2 | 297.0 | 297.1 | 313.0 | 463.1 | 151.8 | 811.1 | 849.2 | 855.3 | 734.2 | 463.7 | 731.7 | 652.9 |
| P44 | 191.8 | 222.1 | 233.5 | 249.2 | 276.6 | 238.2 | 278.4 | 274.8 | 428.6 | 479.6 | 472.7 | 533.4 | 719.5 | 248.8 | 247.6 | 253.3 | 181.9 | 520.5 | 131.1 | 101.7 |

| **Tab. S6** (continued) | | | | | | | | | | | | | | | | | | |
| --- | --- | --- | --- | --- | --- | --- | --- | --- | --- | --- | --- | --- | --- | --- | --- | --- | --- | --- |
|  | P27 | P28 | P29 | P30 | P31 | P32 | P33 | P34 | P35 | P36 | P37 | P38 | P39 | P40 | P41 | P42 | P43 | P44 |
| P27 | 0.0 |  |  |  |  |  |  |  |  |  |  |  |  |  |  |  |  |  |
| P28 | 310.4 | 0.0 |  |  |  |  |  |  |  |  |  |  |  |  |  |  |  |  |
| P29 | 480.1 | 216.9 | 0.0 |  |  |  |  |  |  |  |  |  |  |  |  |  |  |  |
| P30 | 633.3 | 330.3 | 187.4 | 0.0 |  |  |  |  |  |  |  |  |  |  |  |  |  |  |
| P31 | 827.5 | 634.0 | 424.2 | 459.8 | 0.0 |  |  |  |  |  |  |  |  |  |  |  |  |  |
| P32 | 697.3 | 469.9 | 254.4 | 287.6 | 177.5 | 0.0 |  |  |  |  |  |  |  |  |  |  |  |  |
| P33 | 566.7 | 861.0 | 973.8 | 1153.8 | 1180.6 | 1115.6 | 0.0 |  |  |  |  |  |  |  |  |  |  |  |
| P34 | 630.4 | 923.1 | 1031.8 | 1212.9 | 1226.1 | 1167.0 | 64.0 | 0.0 |  |  |  |  |  |  |  |  |  |  |
| P35 | 369.6 | 521.0 | 549.8 | 737.2 | 699.7 | 644.7 | 480.9 | 527.1 | 0.0 |  |  |  |  |  |  |  |  |  |
| P36 | 387.6 | 406.9 | 377.1 | 562.2 | 517.5 | 452.3 | 667.4 | 716.5 | 192.5 | 0.0 |  |  |  |  |  |  |  |  |
| P37 | 338.8 | 337.4 | 323.4 | 510.5 | 522.1 | 433.9 | 682.6 | 735.5 | 227.8 | 69.7 | 0.0 |  |  |  |  |  |  |  |
| P38 | 409.8 | 490.2 | 478.0 | 663.5 | 591.1 | 544.3 | 590.1 | 635.0 | 109.7 | 101.4 | 159.7 | 0.0 |  |  |  |  |  |  |
| P39 | 410.7 | 420.9 | 379.4 | 562.7 | 499.7 | 440.9 | 682.8 | 730.9 | 204.6 | 23.4 | 86.5 | 105.2 | 0.0 |  |  |  |  |  |
| P40 | 808.2 | 702.6 | 531.0 | 633.3 | 240.7 | 355.3 | 1046.1 | 1083.0 | 577.9 | 434.0 | 470.3 | 470.9 | 411.2 | 0.0 |  |  |  |  |
| P41 | 816.5 | 713.8 | 543.2 | 645.9 | 250.8 | 367.7 | 1048.0 | 1084.3 | 581.5 | 440.6 | 478.3 | 475.2 | 417.7 | 12.6 | 0.0 |  |  |  |
| P42 | 717.2 | 586.7 | 409.6 | 514.8 | 182.1 | 245.3 | 1013.4 | 1056.0 | 533.9 | 364.8 | 386.5 | 424.2 | 344.0 | 121.7 | 134.1 | 0.0 |  |  |
| P43 | 726.8 | 606.5 | 433.7 | 542.1 | 200.7 | 273.0 | 1007.2 | 1048.5 | 529.4 | 366.4 | 392.9 | 419.9 | 344.8 | 97.5 | 109.6 | 27.8 | 0.0 |  |
| P44 | 428.7 | 614.2 | 650.5 | 837.8 | 783.0 | 739.0 | 405.6 | 445.3 | 100.7 | 288.9 | 328.3 | 195.3 | 298.2 | 640.6 | 642.7 | 610.9 | 603.2 | 0.0 |
